# Endothelial activation during the diapedesis of cancer cells: between the kiss of death and therapeutic breakthrough

**DOI:** 10.1186/s11658-025-00797-5

**Published:** 2025-10-21

**Authors:** Katarzyna Piwowarczyk, Zbigniew Madeja, Maciej Siedlar, Jarosław Czyż

**Affiliations:** 1 Department of Cell Biology, Faculty of Biochemistry, Biophysics and Biotechnology, Jagiellonian University in Kraków, Ul. Gronostajowa 7, 30-387 Kraków, Poland; 2 Department of Clinical Immunology, Institute of Pediatrics, Jagiellonian University Medical College, Ul. Wielicka 265, 30-663 Kraków, Poland

**Keywords:** Endothelial activation, Diapedesis, Cancer cell transmigration, Endothelial barrier

## Abstract

Cancer development is a complex process, initiated by the combination of epigenetic and genetic changes in normal cells. Selective microenvironmental pressure within the primary tumors prompts the microevolution of invasive cell lineages that efficiently penetrate circulation and lymphatic systems and extravasate in distant organs, initiating the formation of metastases. Extravasation (diapedesis), i.e., the multistep penetration of the endothelial layer by circulating cancer cells, is regarded as the decisive step and one of the bottlenecks of the metastatic cascade. It limits malignant cancer dissemination, while initiating the formation of metastases. The efficiency of extravasation depends equally on the properties of circulating cancer cells and the local functional status of the endothelium, which remains sensitive to paracrine, adhesive, and juxtacrine stimuli generated by cancer and immune cells. Here, we review the current state of knowledge on the significance of endothelial activation for the diapedesis of circulating cancer cells, with the emphasis on the intercellular communication pathways that mediate this process. We also address the potential and limitations of endothelial activation as the target for novel strategies of cancer treatment.

**Graphical Abstract:**

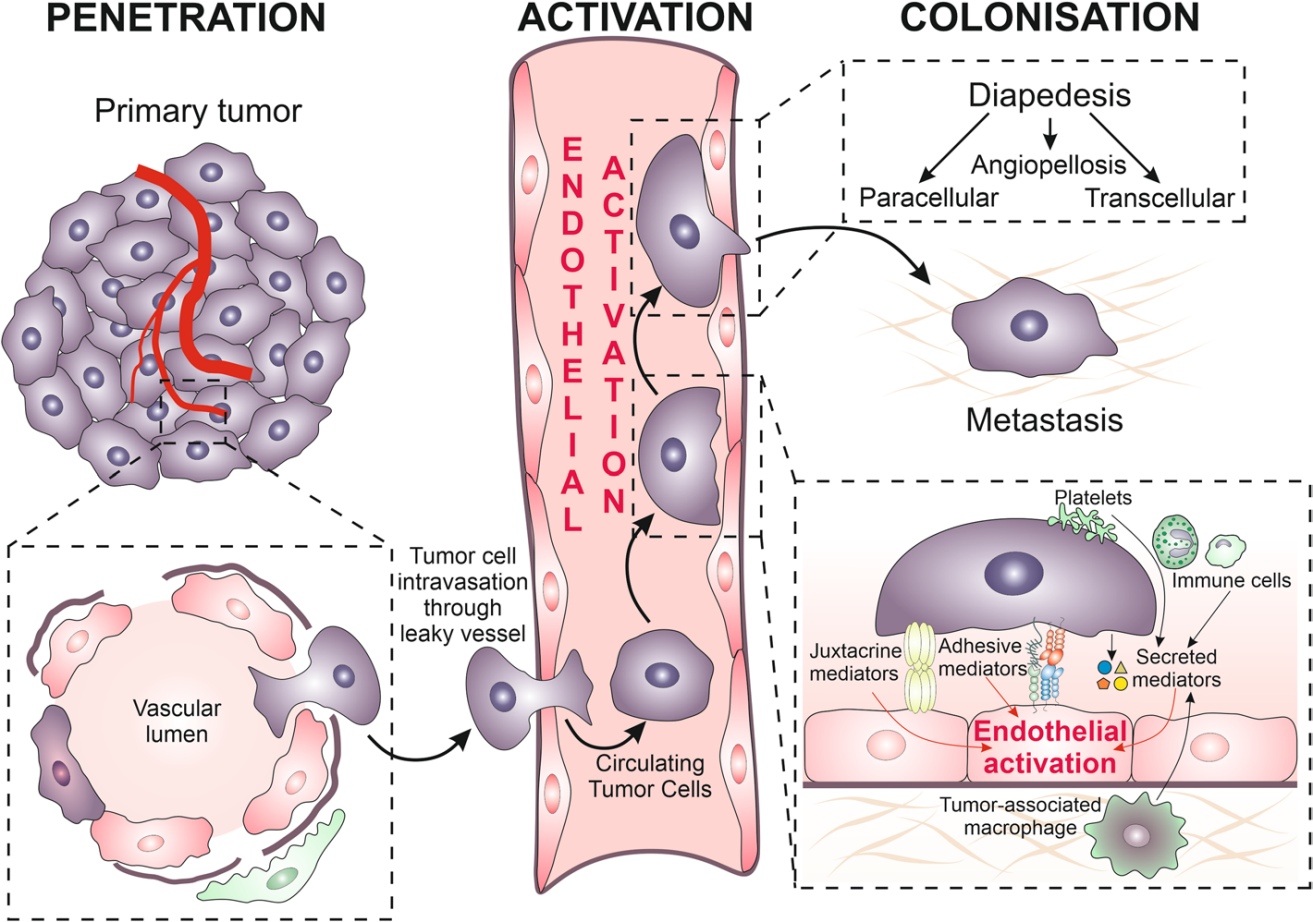

## Introduction

The multidirectional exchange of information between the cells and their microenvironment (niche) is crucial for tissue homeostasis [1]. Its disturbances participate in pathologic processes, including cancer initiation, promotion, and progression [2]. Cancer development is a multistep process, which depends on the combinations of genetic and epigenetic changes in normal cells. The phenotypic “transformation” of tissue cells results in their behavioral imbalance, expansion, rearrangements of local tissue niches, the formation of primary tumor ecosystems, and, finally, metastatic cascade and formation of secondary tumors [3–5]. Meanwhile, the genetic instability of neoplastic cells favors their phenotypic diversification, resulting in the clonal growth of (epi)genetically discrete cell lineages within a heterogeneous tumor cell mass [6]. As the result of consecutive extinction/expansion cycles, invasive cell lineages evolve under microenvironmental pressure to form the invasive front(s). The “clonal evolution” of cancer stem cells (CSCs) [7] cooperates with the continuous adaptation of stromal, immune, and endothelial cells to local niche dynamics. Cancer-associated fibroblasts (CAFs [8]) constitute structural scaffolds for tumor ecosystems, whereas tumor circulation system provides the nutritional and expansion routes for invasive tumor cell lineages. Cancer cells employ versatile invasion strategies to facilitate their migration towards circulation. Local tissue infiltration by tumor cells is facilitated by their expansive growth, ameboid and mesenchymal migration of individual cells, and the collective cell movement [9]. It is directed by an array of chemotactic/haptotactic cues generated by stromal and immune cells and the extracellular matrix. Due to the impaired barrier properties of tumor circulation, cancer cells can relatively easily infiltrate this system (both blood and lymphatic vessels) [10, 11]. Generally, larger lumens and the lack of continuous basement membrane make lymphatic vessels easier to infiltrate by cancer cells than blood vessels. Thus, lymphatic vessels provide cancer cells with a comfortable route for their systemic dissipation. The subsequent entry of cancer cells into the blood system helps them to evade immune defenses and opens the route for the colonization of distant organs. In turn, effective endothelial barriers of the blood system in healthy tissues limit the efficiency of this process, constituting an important checkpoint for the formation of metastatic loci.

Diapedesis has long been considered in terms of the properties of circulating cancer cells; however, nowadays it has become clear that its efficiency equally depends on the activation of adjacent endothelium. It is initiated by the adhesion of cancer and endothelial cells, followed by the disruption and penetration of endothelium, which results in the gestation of cancer cells in supraendothelial layers. Thus, the efficiency of extravasation relies on the local impairment of the endothelial barrier function. The local activation of endothelial cells induces their contraction and the disintegration of the whole endothelial layer, facilitating the penetration of the endothelium and underlying structures by circulating cancer cells [12, 13]. The coordination of these processes is ascertained by complex communication systems that mediate the exchange of information between endothelium and cancer cells [14–20].

Extravasation is a critical and decisive step in the metastatic cascade that determines whether a circulating tumor cell (CTC) fails or succeeds in forming metastasis. It is estimated that only < 0.1% of circulating cancer cells continue to form secondary tumors [21]. Thus, extravasation can be seen as the “kiss of death” for most tumor cells, but the rare ones that go through this step give the “kiss of death” to the organism by starting deadly colonies. Due to the role of extravasation in the metastatic cascade, the identification of mechanisms underlying the active contribution of endothelium to this process is of utmost importance for the identification of mechanisms underlying tumor mortality. As one of the bottlenecks of the metastatic cascade, it can also serve as a target for novel cancer treatment regimens that may potentially lead to “therapeutic breakthroughs.” Here, we summarize the state of the art on (i) the role of endothelial activation in the cancer cell diapedesis as key point in the metastatic cascade and (ii) the significance of “premetastatic niches” for this process. Special emphasis is focused on (iii) the mediators of endothelial activation in “premetastatic niches.” Finally, (iv) we provide an overview of the prospects and limitations related to the pharmacological targeting of endothelial activation in cancer therapy.

## Endothelial barrier function

The endothelium is an efficient but highly selective barrier, which separates cells and molecules circulating in the blood vessels from the solid tissue microenvironments. Endothelial cells that line the interior of blood vessels actively mediate the exchange of chemicals between the circulation system and tissues. Endothelial integrity is ascertained by intercellular junctions, which bind adjacent endothelial cells along the entire length of the cell’s contact with its neighbors [22–24]. The dynamics of these junctions regulate cell–cell adhesion, paracellular permeability, and signal transduction, all of which are crucial for vascular homeostasis and responsiveness to physiological and pathological stimuli. Three major types of endothelial cell junctions—tight junctions, adherens junctions, and gap junctions—cooperate in preservation of endothelial barrier function. Among them, adherens junctions play a key role in maintaining vascular integrity, permeability and remodeling of the vascular walls. They are stabilized by VE-cadherin-mediated homophilic interactions. Adherens junctions link the cytoskeletal systems of neighbor cells via linker complexes comprising an array of proteins, including β-catenin, p120-catenin, and α-catenin [25]. Apart from their structural function, adherens junctions determine endothelial cell reactivity to physical stimuli (including shear stress) and inflammatory signals, thereby adjusting endothelial permeability and limiting leukocyte transmigration [22]. The disruption of adherens junctions, whether through internalization of VE-cadherin or changes in the mechanical equilibrium of the cytoskeleton, can lead to increased vascular leakage and contributes to pathological conditions such as sepsis or cancer cell diapedesis [23, 26, 27].

Furthermore, endothelial barrier function is determined by tight junctions, which limit the passage of ions, solutes, and immune cells between the bloodstream and surrounding tissues. These structures consist of a number of transmembrane proteins, including claudins, occludin, and junctional adhesion molecules (JAMs). They interact with cytoplasmic scaffold proteins such as ZO-1, ZO-2, and ZO-3, linking tight junction complexes to the actin cytoskeleton. In the endothelium, tight junctions are especially well developed in barrier-forming vessels, such as brain vessels, where a strict control of permeability is crucial for neural tissue integrity and protection. They help maintain vascular homeostasis by limiting transendothelial leakage and the passage of immune cells and pathogens and by preserving endothelial cell polarity and structural integrity [28]. In turn, direct intercellular communication between neighboring endothelial cells is accomplished via the clusters of aqueous intercellular gap junctional channels. Gap junctions are built of connexins and mediate the direct intercellular diffusion of small (< 1.5 kDa) metabolites (gap junctional intercellular coupling; GJIC). A total of six connexin molecules form a hexamer (hemichannel; connexon) that docks to its counterpart built into the membrane of neighbor cells to form the channel. GJIC participates in the electrical and metabolic cell coupling of the cells within tissues. The disruption of these intercellular junctions can result in increased vascular permeability, inflammation and contributes to the pathogenesis of tumor metastasis, as well as various cardiovascular and inflammatory diseases [29–32]. Collectively, adherens junctions, tight junctions, and gap junctions form an integrated system that determines the barrier function of endothelia and acts as a dynamic regulator of vascular physiology [22, 24, 26, 28].

**Fig. 1:**
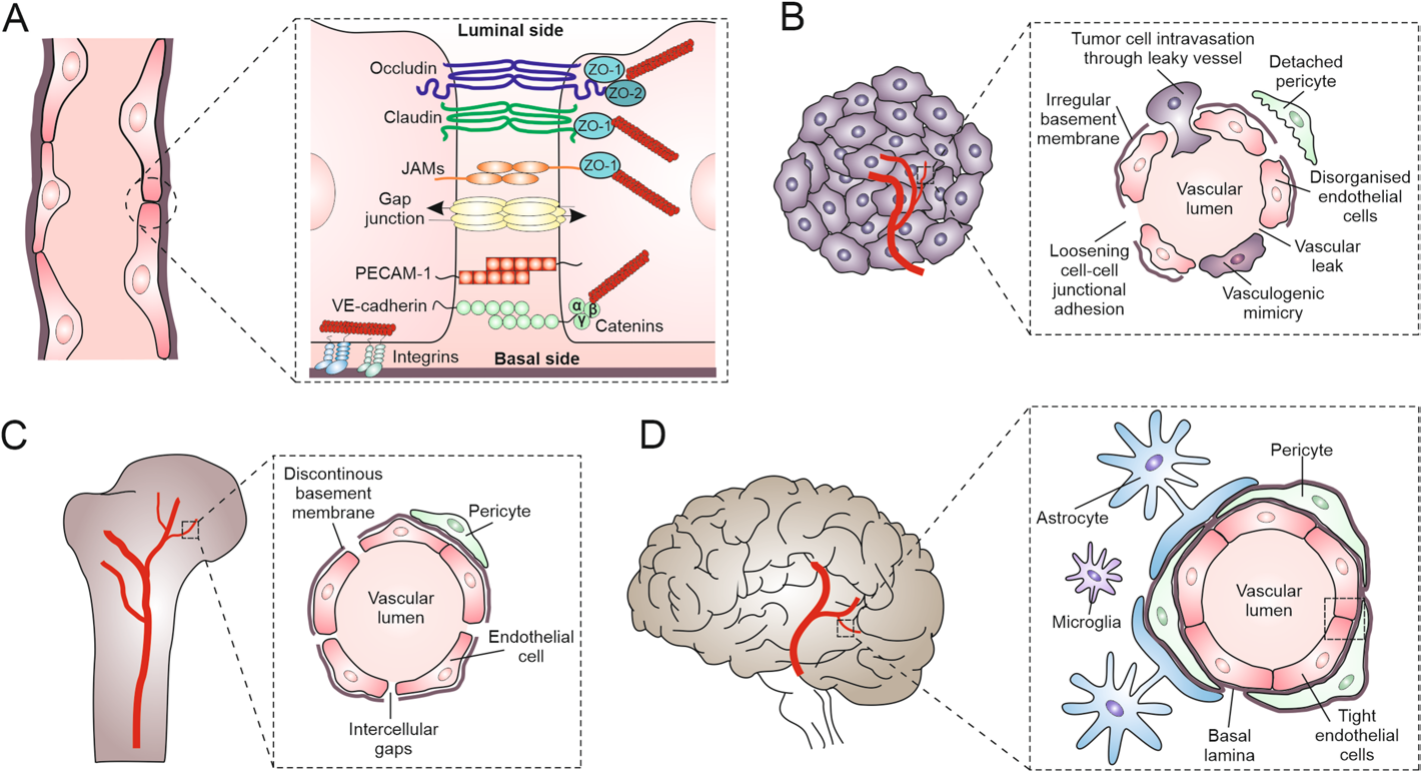
Structural characteristics and key molecular components of blood vessels, in relation to their localization. **A** Membrane receptors critical for maintaining the normal vessel integrity (tight junction proteins (occludin, claudin, JAMs), adhesion molecules (PECAM-1, VE-cadherin), gap junctions, and catenins). **B** Structure of tumor-associated vasculature, with disorganized endothelial layers, irregular basement membranes, detached pericytes, and vasculogenic mimicry (a process by which aggressive cancer cells form blood-vessel-like structures without involving endothelial cells, allowing tumors to obtain nutrients and oxygen independently of normal blood vessels). Loosened cell–cell adhesion complexes allow vascular leakiness and facilitate tumor cell intravasation. **C** A discontinuous basement membrane and intercellular gaps between endothelial cells, accompanied by unstructured pericyte layers in bone. **D** Solid organization of brain vessels, including endothelial cells, pericytes, basal lamina, and astrocytes, ascertain their efficient blood–brain barrier (BBB) function. Figure prepared using CorelDRAW Graphics Suite 2020 (Corel Corporation, Ottawa, Canada). Note the tissue-specific structural variability of blood vessels

The endothelial lining of blood/lymphatic vessels is surrounded by pericytes, characteristic for microvessels, or smooth muscle cells that form protective layers around the veins and arteries [33]. Due to their contractile activity, perivascular cells also participate in the regulation of blood pressure, stabilizing the permeability of endothelium. As such, they determine their physical stability, constitute an additional barrier for extravasating cells, and participate in the neoangiogenesis [34]. Local properties of endothelium vary between tissues and organs (Fig. 1). They depend not only on the vessel size (micro-/microvasculature) or type (veins versus arteries) but also on the tissue type or origin (blood–brain barrier versus lymphatic vessels; excellently reviewed by Aird et al. [33]). For example, endothelial cells in the brain and bone differ significantly in structure and function owing to the unique requirements of their environments [26, 27, 35]. The brain endothelial cells form the highly selective blood–brain barrier (BBB), which is characterized by dense tight junctions. Abundance of membrane transporters in brain endothelium protects neural tissue from toxins and regulates transendothelial trafficking of small molecules [36]. In turn, bone endothelial cells are more permeable, with looser junctions and, in some cases, the fenestrations that facilitate nutrient exchange and support bone remodeling. While the brain endothelium is closely associated with astrocytes and pericytes to maintain strict barrier control, bone endothelium interacts with less structured pericytes. Tissue-specific phenotypes of endothelial cells further affect their structural and functional dynamics, including the susceptibility of endothelial layers to the activating signals generated by immune, cancer, and/or stromal cells [34, 37, 38].

The multistep penetration of the endothelial layers by circulating cells remains a crucial point of numerous physiological and pathological processes, including the inflammation and tissue regeneration [39]. The knowledge on the mechanisms determining the local reactivity of endothelium to the signals from cancer cells originates from the studies on the diapedesis of immune cells [18, 40]. These studies identified an array of pathways involved in this process and in the regulation of endothelial barrier function as two opposite forces that determine the efficiency of immune cell extravasation. Circulating immune and cancer cells employ corresponding mechanisms of extravasation [20]; however, there are also obvious differences, which are related to the size, mechanical deformability, dynamics of secretome, and adhesiveness of cancer cells and affect the pattern of their diapedesis. Discrimination of the common and cell-type-specific principles that govern endothelial activation during the diapedesis of circulating immune and tumor cells is crucial for elaboration of the therapeutic regimens that specifically target the diapedesis of cancer cells without exerting side effects on the function of the immune system.

## Diapedesis of immune cells

Immune cell diapedesis is a physiological example of the highly specific and selective cellular penetration of endothelial barriers. This process is relatively quick and usually takes about 2 h [41]. Its individual steps are reversible; however, the completed transendothelial migration of immune cells is considered a “point of no return” in the process of inflammation. A number of pioneering works outlined the principles of leukocyte homing and arrest in inflamed tissues (reviewed in [42, 43]). Normally, it is prompted by the local inflammatory response of the tissue cells to damages and/or pathogens; however numerous cofactors affect the efficiency of immune cell diapedesis. It directly depends on the functional status of endothelium, the type of immune cells, and the intensity of inflammation stimuli [44–46]. Other key factors crucial for the immune extravasation cascade include the signals exchanged between stromal, endothelial, and circulating immune systems that commit immune cells to extravasate and invade the inflamed tissue. Despite this diversity, the principal stages of immune cell diapedesis remain universal.

During the first step of immune extravasation, stromal cells mobilized by local microenvironmental cues start to secrete proinflammatory cytokines (for instance, interleukins (IL)-1, -6, -8, -12, and tumor necrosis factor alpha (TNF-α)) to locally activate the endothelial cells. The upregulation of cell adhesion molecules (CAMs) results in their accumulation on the apical surfaces of endothelial layers, facilitating the “trapping” of leukocytes from the bloodstream around the site of inflammation. During this process, endothelial P-, L-, and E-selectins can interact with mucin glycoprotein receptors (PSGL-1) or sialyl Lewis X-/A-containing glycoproteins, which are present on the surfaces of leukocytes [42, 47]. They cooperate with integrins (e.g., α_L_β_2_ (LFA-1), α_M_β_2_ (Mac-1), and α_4_β_1_ (VLA-1)) in the low-affinity binding of leukocytes to the endothelium that promotes the “rolling” of leukocytes along the blood vessel wall [48, 49]. It is followed by their stable adhesion to the endothelium via the receptors of higher binding strength [50, 51]. A feedback loop of interleukin-dependent intercellular signaling, apical CAM accumulation, adhesion of rolling immune cells, and stable adhesion between the partner cells provides the background for the active participation of endothelium in the “paracellular” and “transcellular” diapedesis of leukocytes.

The paracellular route of transendothelial migration (TEM) is a predominant mechanism of immune cell diapedesis. During this process, selectin-mediated rolling, chemokine-triggered activation, and integrin-dependent arrest is followed by the morphological polarization of stably adhered leukocytes. Consequently, they acquire the ability to move over endothelial surfaces in search of the sites permissive for TEM [52]. During its paracellular variant, the sequential activation of adhesion receptors on the surfaces of immune and endothelial cells prompts the local impairment of endothelial integrity. The disengagement of specialized subcellular structures (lateral border recycling compartment—LBRC), localized at the intercellular contact areas of endothelial layer, cooperates with the activity of podosomes of immune cells to loosen intercellular junctions in endothelia and facilitate leukocyte diapedesis [44, 53]. Podosomes, as labile membrane protrusions, squeeze through the contacts between endothelial cells pulling the rest of the cell body behind [54] via sequential interaction of their surface receptors with endothelial PECAM-1, JAM-A/C or CD99 [45, 48]. It begins with ICAM-1 accumulation in endothelial cell membranes and its interaction with CD11/CD18 present on leukocyte surfaces [55]. The consequent activation of intraendothelial signaling pathways leads to the local VE-cadherin disengagement and endothelial barrier disruption [23]. Collectively, endothelial activation is necessary for the effective paracellular TEM of immune cells.

An alternative (transcellular) mode of immune extravasation is based on the direct transmigration of immune cells through endothelial cell bodies [46, 48]. During the transcellular diapedesis, leukocyte podosomes generate forces that probe endothelial surfaces, causing deep membrane invagination in the site of prospective diapedesis [52]. It brings together the apical and basal endothelial cell membranes, leading to the transcellular pore formation without the disruption of the intercellular adhesion complexes within the endothelial layer [56, 57]. Transcellular diapedesis of immune cells depends on the interplay between paracrine signaling and cell adhesion receptors. During the transcellular diapedesis of T lymphocytes, a long-term activation of TNF-α signaling has been shown to cooperate with the ICAM-1 mobilization on endothelial surfaces. Other surface receptors involved in this process include: PECAM-1, CD-99, and JAM-A as well as the LBRC compartment [58]. In addition, caveolin-1 is accumulated at the sites of transcellular diapedesis [59], whereas its downregulation may favor the paracellular transmigration mode [60]. In turn, Mac-1 negatively regulates this process in leukocytes [49, 51, 61]. Collectively, the loops established between stromal, endothelial, and immune cells locally activate endothelial cells to facilitate the transcellular diapedesis of leukocytes. Consequently, endothelial cells appear as active players during the extravasation of immune cells.

After passing through the endothelium, leukocytes can invade the outer vascular layers and adjacent stroma. Predominantly, this process relies on their ameboid movement, which is based on the high deformability of immune cells and directed by the gradients of chemoattractive cytokines. Numerous cytokines act as chemotactic factors for immune cells. For instance, CSF-1 and MCP-1 act as chemoattractants for monocytes/macrophages [62, 63], whereas CXCL1–CXCL-3, CXCL5–CXCL8, chemotactic lipids, complement anaphylatoxins, and formyl peptides induce the chemotaxis of neutrophiles [64]. They participate in the local paracrine loops that collectively control the movement of immune and cancer cells during the invasive front formation and metastasis [65–68]. For instance, breast cancer cells secrete CSF-1 that stimulates the movement of macrophages into the tumor milieu. Macrophages form the gradient of epidermal growth factor (EGF) between the vessels and tumor niches that prompts the migration of tumor cells towards the vessels [69]. Corresponding two-directional cellular highways, which are potentially involved in cancer progression, have been described elsewhere [70, 71]. For instance, activated macrophages secrete TNF-α that acts as a stimulator of motility and proinvasive phenotypic transitions for pancreatic cancer cells [72]. Such loops are also established in the premetastatic niches, where they promote the establishment of secondary tumor ecosystems. Collectively, cooperative paracrine and adhesive systems participate in the establishment of the signaling loops between endothelial, extravasating immune, and cancer cells that promote the formation of secondary tumor ecosystems. They illustrate the significance of endothelial cell activation for this process.

## Tumor cell diapedesis

The “seed and soil” theory, proposed by Stephen Paget in 1889, predicts that metastasis depends equally on the characteristics of cancer cells (the “seeds”) and on the properties of the target organ microenvironments (the “soils”) [4]. The shaping of cancer cell phenotype starts already in primary tumor niches, where the disturbances of local vasculature induce nutritional deficits, hypoxia and the accumulation of toxins in the tissues. Intoxication of resident cancer cells can prompt their (epi)genetic instability, transformation, and the initiation of cancer development [73]. As mentioned above, paracrine loops between neutrophiles, lymphocytes, tumor-associated macrophages (TAMs), and cancer cells increase their ability to penetrate peritumoral niches in a manner dependent on TAM-secreted EGF [69] and TNF-α [72]. At later stages, in the case of EGF, this effect can be executed via the Mena^INV^-mediated cytoskeleton remodeling, which induces the formation of invadosomes by cancer cells and enhances their invasive potential [74, 75]. These invasive “seeds” reciprocally modify the “soils” of primary tumors. In particular, they induce continual vascular rearrangements and the continuous activation of tumor endothelial cells. Disorganization of intratumoral vessels (excellently reviewed by [34, 76–79]) provides the background for further primary tumor progression [34, 80–82] and for the initiation of metastatic cascade. In particular, the lack of continuous basal laminae and peripheral layers impair their barrier functions, facilitates the intravasation of cancer cells and their further systemic dissemination [83]. This notion is confirmed by the beneficial effects of the normalization of intratumoral vasculature, which interferes with the expansion of primary tumors and the formation of invasive front(s) [84, 85].

**Fig. 2:**
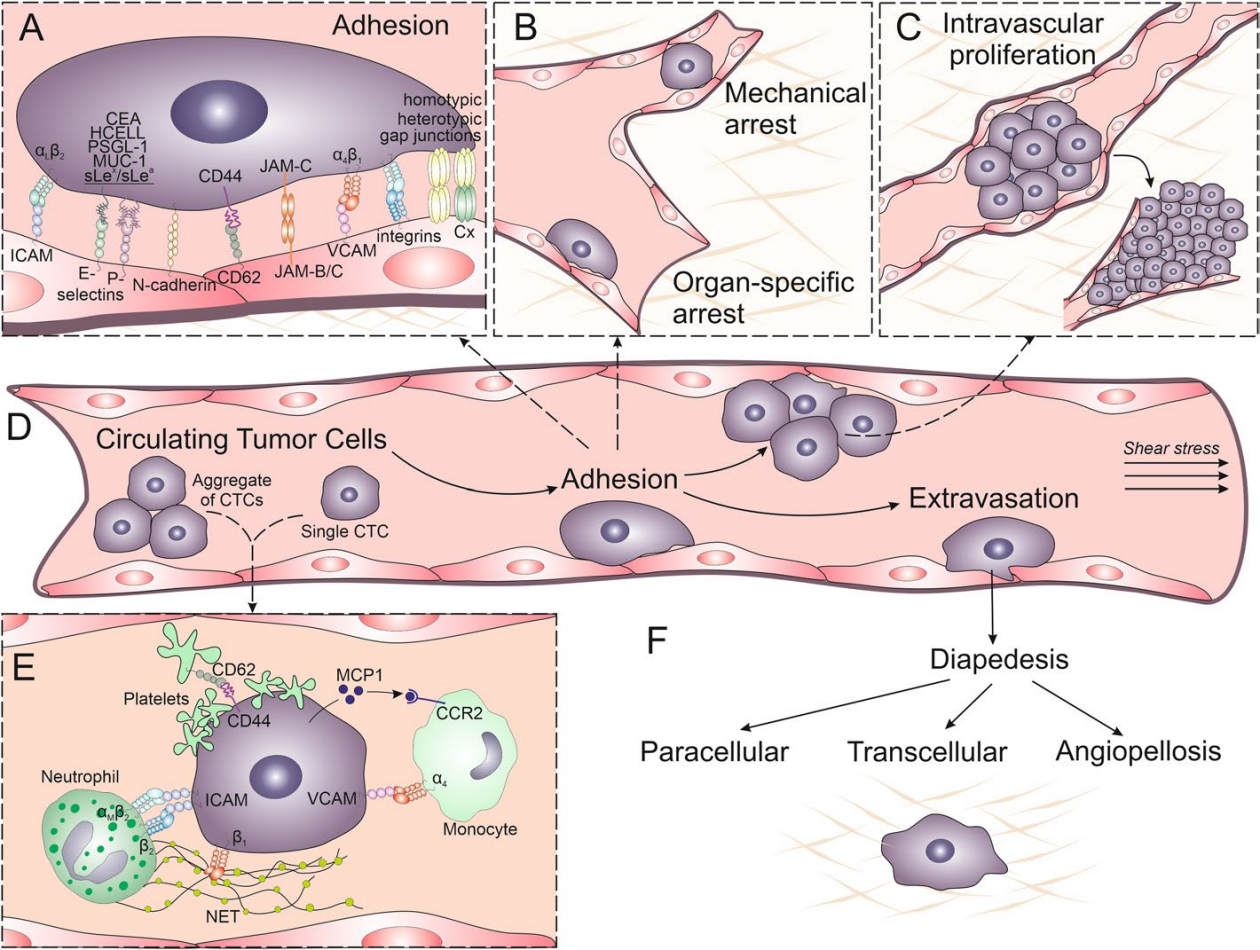
Processes underlying adhesion, vascular arrest, and extravasation of circulating tumor cells (CTCs). CTCs utilize an array of cell adhesion receptors, including integrins, CD44, ICAM, VCAM, and selectins to adhere to endothelial cells. Homotypic or heterotypic gap junctions mediate the exchange of signals that augment adhesive competence of CTCs and local endothelium (**A**). Concomitantly, CTCs recruit neutrophils, monocytes, and platelets. Platelets present CD62, promoting CTC adhesion, while neutrophil extracellular traps (NETs) facilitate CTC capture in premetastatic niches (**E**). Following the organ-specific adhesion of single CTCs (**B**) and their aggregates to endothelium (**D**) or mechanical arrest and intravascular proliferation of CTCs in narrow capillaries (**B**, **C**), resulting in the formation of aggregates, single CTCs or their clusters exit the vasculature through paracellular (junctional), transcellular (through cell bodies), or angiopellotic variants of diapedesis (**F**). Figure prepared using CorelDRAW Graphics Suite 2020 (Corel Corporation, Ottawa, Canada)

To complete the diapedesis and homing in the healthy tissue, circulating tumor cells (CTCs) adapt an array of mechanisms, which are usually employed by immune cells. Whereas the “transcellular” strategy of CTC extravasation is observed rather rarely [86], CTCs predominantly penetrate endothelial barriers at the sites of the contact between adjacent endothelial cells. It means that the paracellular strategy is a basic mechanism of cancer cell extravasation, even though CTCs may also proliferate intraluminously [87]. Notably, the size of cancer cells can facilitate this process, as it prompts their immobilization and aggregation in capillaries. However, it is also observed in capillaries of internal diameter larger than the diameters of tumor cells (including precapillary arterioles of the lung [87]). Collectively, these strategies provide the basis for (i) the arrest of CTCs within microcapillaries, followed by (ii) “paracellular” diapedesis and (iii) the angiopellotic strategy of extravasation (Fig. 2). They are accompanied by the concomitant formulation of “premetastatic niches,” which facilitates cancer cell gestation in distant organs.

### Formation of premetastatic niches during tumor metastasis

Whereas the circulation system constitutes a route for the cancer “metastatic cascade” and the development of secondary tumors, it also exerts selective forces on circulating cancer cells. As the consequence of cytotoxic activity of immune cells [88], the anoikis (due to loss of integrin-dependent adhesion) [89] and the hydrodynamic shear stress/mechanical deformations within the microcapillaries [4, 90], CTCs can survive in bloodstream for only a limited period of time after intravasation. Therefore, CTC “survivors” represent an elite of stress-resistance, capable of adapting to hostile niches. Perhaps, therefore, the numbers of CTCs detected in the circulation of patients with advanced tumors are relatively low compared with white blood cells (1–10 cells per mL of blood) [91]. Next, the intact structure and barrier function of endothelia within healthy tissues implies the limiting role of endothelial activation during the metastatic cascade [92]. It explains why the extravasation is an important limiting factor during the metastatic cascade [93, 94]. Actually, despite considerable cancer cell survival in the bloodstream, only less than 0.1% of circulating cancer cells form secondary tumors [21, 93]. By contrast, accumulating evidence suggests that primary tumors actively prepare distant tissue niches for metastatic colonization by CTCs, whereas their welfare in the circulating system remains protected by immune cells.

Systemic signaling [95, 96], which determines the formation of “premetastatic niches” (PMNs), is mediated by immune cells [96, 97], extracellular matrix components [96, 98], signaling molecules [96, 98–104], and exosomes [105]. Cancer cells, which infiltrate adjacent primary tumor stroma and intravasate, secrete a variety of signaling molecules including cytokines (TNF-α [100], IL-6 [101], IL-1β [102]), chemokines (CCL2 [98]), growth factors (vascular endothelial growth factor (VEGF) [99, 103], TGF-β [104]), and extracellular vesicles [105]. These tumor-derived signals mediate long-range communication, triggering molecular and cellular changes in distant organs. They prompt the recruitment and mobilization of bone-marrow-derived cells (BMDCs), including myeloid-derived suppressor cells (MDSCs), macrophages, neutrophils, and endothelial progenitor cells [96, 106], which contribute to local immunosuppression, neoangiogenesis, and extracellular matrix (ECM) remodeling, creating conditions favorable for subsequent tumor cell colonization. ECM reorganization in premetastatic niche can be induced by tumor cells-secreted enzymes, such as lysyl oxidase-like 2 (LOXL_2_), which reciprocally induce CXCL12 expression in local fibroblasts and enhance cancer cell migration and PMN tropism [98]. Concomitantly, increased vascular permeability and local inflammation further facilitate extravasation, gestation, and early survival of CTCs in a distant site.

### Capillary arrest and paracellular diapedesis of cancer cells

CTCs use an array of strategies that help them to negotiate endothelial barriers, extravasate, and initialize the formation of metastasis. They are partly similar to that adapted by immune cells; however, they also differ at several important points (Table 1) [107]. These differences are enforced by the different properties of immune and cancer cells. Relatively small dimensions and high mechanical elasticity predestine immune cells to survive within highly a dynamic microenvironment of blood/lymphatic system. Immune cells usually exploit an ameboid strategy of migration, which enables extensive deformations without broad cytoskeletal reorganization. The absence of nuclear lamins further enhances the nuclear deformability of immune cells, adding to their transmigration capacity [108]. Some cancer cells are also characterized by high viscoelastic properties, which help them to reshape their bodies and nuclei and to undergo a mechanical deformation during microcapillary transit [90, 109, 110]..

**Table 1:** Comparison of characteristic features of immune and cancer cell diapedesis through endothelium barrier

	Diapedesis	
	Immune cells	Cancer cells
Process	Inflammation or immune surveillance	Cancer progression
Driver	Tissue injury or infection	Local inflammation/autocrine loops
Transmigration route	Largely ameboid Paracellular and transcellular	Largely mesenchymal Mainly paracellular; also transcellular, angiopellosis, or intraluminous proliferation
Speed	Fast	Slow
Proteolysis	Minor protease activity	Major protease activity Degraded basement membranes
Endothelial disruption	Limited in time and space	Prolonged
Outcome	Immune cell infiltration	Gestation of tumor cells
Consequences	Physiologic: maintenance of tissue homeostasis	Pathological: metastasis

Interestingly, the extravasation of cancer cells can be regulated by the fluidity of cell membranes. Wang et al. (2024) have shown that the increased incorporation of polyunsaturated fatty acids (PUFAs) into cell membrane lipids, which is driven by acyl-coenzyme A synthetase long-chain family member 4 (ACSL4), enhances membrane fluidity and metastatic cell extravasation [111]. Enzymes involved in designating unsaturated fatty acids for β-oxidation (such as ECH1) may participate in this process, linking metastatic potential of cancer cells with their metabolic profile. Consequently, targeting this process through coinhibition of ACSL4 and ECH1, may represent a promising therapeutic strategy. By contrast, cancer cells are usually much bigger in size than their immune counterparts. They are also less susceptible to shear forces and deformations resulting from squeezing through narrow microcapillaries [90, 112, 113]. The low mechanical elasticity of these cells impairs their transendothelial migration and implies the contribution of activated endothelia to facilitate this process.

In 1928, James Ewing concluded that relatively large diameters of cancer cells predestine these cells to the mechanical trapping in the narrow capillaries of circulatory system [114]. Nowadays, there is a general consensus that the capillary arrest of single and/or aggregated CTCs commonly initiates the extravasation of cancer cells [115, 116]. The mechanical arrest of CTCs facilitates the direct crosstalk between the cancer cells and endothelium, the activation of endothelial cells, disruption of endothelial integrity, and paracellular CTC diapedesis. As its consequence, arrested tumor cells can migrate against blood flow within the narrow vessel lumens to survey the suitable and favorable site for extravasation (i.e., the “premetastatic niche” [117, 118]). Additionally, the microaggregates of cancer cells with the immune cells (leukocytes) and platelets [119–121] work as the shields, protecting circulating cancer cells from excessive physical forces and immune responses, and prompting the local mobilization of endothelia [122, 123]. In turn, the “rolling” of CTCs over endothelial layers is observed relatively rarely [124–126]. However, it has been shown in prostate cancer, breast cancer, and melanoma models. E-selectin, N-cadherin, integrins, and immunoglobulin-like adhesion molecules participate in this process [20, 82, 124, 127–129]. Notably, E-selectin accumulation on the surfaces of endothelial cells can be induced by proinflammatory cytokines, secreted by cancer cells or macrophages [130, 131]. Regardless of the mechanism underlying the stable endothelial adhesion of CTCs, endothelia and immune cells cooperatively determine the preferred formation of metastases within the organs, which are rich in small and sinusoidal capillaries (such as the lung, bones, bone marrow, and liver), as predicted by the seed and soil hypothesis [116, 132–135].

**Fig. 3:**
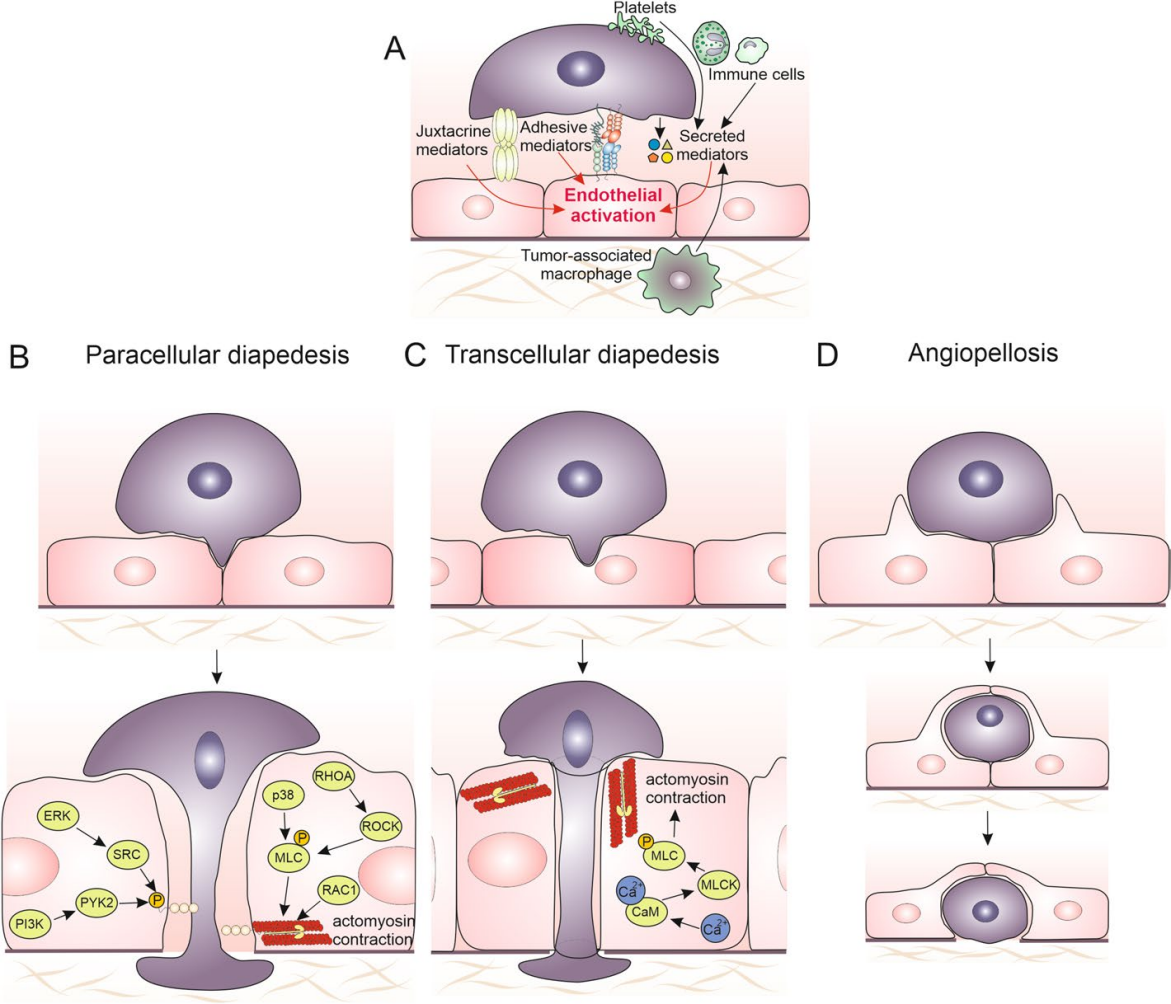
Pathways of tumor cell extravasation. Diapedesis is the result of the activity of at least two systems: vasoactive factors secreted in the metastatic niche and direct contact between the endothelium and CTCs via the adhesion proteins present on the surface of both types of cells (**A**). Tumor cells can traverse between endothelial cells, disrupting intercellular junctions (**B**). This process involves a range of signaling pathways, including PI3K, SRC, ERK, and Rho GTPases, which activate actomyosin contraction to open endothelial gaps. Proteins such as myosin light chain (MLC) and p38 kinase mediate the cytoskeletal reorganization necessary for passage of CTCs through endothelial barriers. Alternatively, CTCs pass directly through endothelial cells, creating a transcellular pore (**C**). This process is facilitated by actomyosin contraction and signaling involving myosin light-chain kinase (MLCK), calmodulin (CaM), and RhoA pathways, which reorganize the endothelial actin cytoskeleton to open the transcellular pore. Finally, CTCs are passively extruded from the vessel via coordinated remodeling of microcapillary walls (**D**). Endothelial cells form a “pocket” around the tumor cells to expel them into the surrounding tissue without significant disruption of junctional integrity. Figure prepared using CorelDRAW Graphics Suite 2020 (Corel Corporation, Ottawa, Canada). Note the diversity of tumor extravasation mechanisms, showing the active role of endothelium in the processes initiating the formation of metastases

Endothelial activation is also crucial for the later stages of CTC diapedesis, when the formation of cancer-specific adhesive invadopodia determines the paracellular diapedesis of CTCs (Fig. 3). Mature invadopodia are long-lived, shallow cell protrusions that interact with the extracellular matrix (ECM) [118, 136]. They comprise F-actin core accompanied by the structural proteins (cortactin, Tks4, Tks5, N-WASp, Arp2/3) [136–138]. As such, these structures are similar to the immune cell podosomes. During the diapedesis of cancer cells, they dynamically interact with the endothelium, causing the disruption of their continuity at the intercellular junctions [118]. It is accompanied by the changes in the profile of their surface receptors and by the reorganization of the endothelial cytoskeleton. Consequently, the activity of invadopodia cooperates with the reorganization of endothelial adhesion complexes to locally impair endothelial barrier function. Thus, their formation is correlated with the highly invasive cell phenotypes [75, 137] and facilitates the diapedesis of cancer cells [137, 139].

### Angiopellosis

Alternative mechanisms, which establish structural platforms for subsequent CTC extravasation [140], include the formation of multicellular aggregates and chains. They further optimize the mechanical deformations of cancer within capillary blood vessels [141] and protect CTCs against the hostile bloodstream environment. An ultimate example of diapedesis scenario that depends on combined CTC aggregation and endothelial activation is provided by angiopellosis (*angio*: vessel-related; *pello*: drive out; [142]). In contrast to other scenarios of diapedesis, which are shared by immune and cancer cells, angiopellosis was described as an extravasation mechanism of CTC clusters. They can exploit this strategy to overcome endothelial barriers during the metastatic cascade [143]. Similarly to other extravasation variants, this multistep process is initiated by the adhesion of CTCs to the vessel walls. Subsequently, the adjacent endothelial cells extend membrane protrusions to form an intraluminal “pocket” covering up the extravasating cells and/or their aggregates. It is followed by an active expulsion of cancer cell aggregates to parenchyma. The collective mechanical activity of endothelial cells plays a crucial role in this process. Active vascular expulsion and endothelial pocketing were also described for the cardiac and mesenchymal stem cells. Whereas its significance for inflammatory processes and metastatic cascade remains to be fully elucidated, different mediators are conceivably involved in the angiopellosis and paracellular diapedesis of cancer cells [142]. Collectively, phenotypes and tissue-specific properties of CTCs and endothelial cells, respectively, determine the pattern of CTC diapedesis. Their various combinations account for the predilection of tumors to form metastases in specific organs. Regardless of the CTC/endothelial phenotype, the efficiency of this process is directly related to endothelial activation and depends on the susceptibility of endothelial cells to the signals generated by immune cells and CTCs.

### Contribution of immune system to the diapedesis of cancer cells

Regardless of the underlying mechanism(s), CTC extravasation pattern is modified by immune cells. Neutrophils form physical aggregates with CTCs via β_2_ integrin-ICAM-1 interactions, enhancing the arrest of CTCs on the endothelium [144]. Next, they suppress natural killer (NK) cells and support tumor cell extravasation in a IL-1β- and MMP-dependent manner [145]. In turn, CXCL chemokines secreted by CTCs induce the release of neutrophil extracellular traps (NETs) by neutrophils and granulocytic myeloid-derived suppressor cells (MDSCs) [146]. NETs protect CTCs from the cytotoxic activity of CD8⁺ T lymphocytes and NK cells [146] and physically sequester CTCs via β_1_-integrin mediated interactions [147]. These processes enhance the efficiency of CTC extravasation but also increase endothelial permeability in a manner dependent on reactive oxygen species (ROS), matrix metalloproteinase 9 (MMP9) [148], and proinflammatory cytokines (TNF-α, IL-1β, and IL-6) [149, 150]. They also upregulate adhesion molecules such as ICAM-1, VCAM-1, and E-selectin, thereby enhancing tumor cell adhesion under shear flow conditions [151]. Collectively, immune cells are not passive bystanders but active facilitators of tumor cell extravasation. Through modulation of CTC and endothelial cell properties, these cells significantly contribute to metastatic progression.

## Hallmarks of endothelial activation during the diapedesis of cancer cells

Since the endothelium plays a direct role in the diapedesis of leukocytes and neoplastic cells, extensive studies were performed to trace the hallmarks of endothelial activation during this process. The activation of endothelial cells CTCs at the site of diapedesis is determined by both the intrinsic properties of the tumor cells and the functional status of the local endothelium. A range of general hallmarks of this process has been identified. First, the local remodeling of microcapillaries follows the stabilization of CTC adhesion to endothelial layer in “premetastatic niches” [40]. Next, at the biomechanical level, endothelial activation is accompanied by cytoskeletal rearrangements that result in the increased contractility of endothelial cells. They facilitate the disruption of intercellular contacts, due to the retractive activity of endothelial cells and/or the active demobilization of cell adhesion apparatus at intercellular interfaces. At places, an induction of endothelial cell motility and proliferation is observed. Contraction of endothelial cells adjacent to their cancer counterparts is illustrated by the formation of microfilament bundles (stress fibers) and the maturation of focal adhesions at the regions of endothelium adjacent to cancer cells [152]. This effect is accompanied by the induction of endothelial cell movement [15]. Notably, endothelial cells can also react to the activating stimuli with the induction of apoptosis/necroptosis [153]. They collectively impair local endothelial barrier function in the proximity of CTCs in a manner dependent on the tissue-specific properties of endothelial cells in various organs [26, 35, 154]. Due to their relationship to neoangiogenesis, endothelial activation prospectively opens the windows for the neovascularization of secondary tumor ecosystems. Its significance prompted the development of techniques aimed at the identification of the mediators of this process, their tissue-specificity, and the potential for therapeutic targeting.

### Modeling endothelial activation in vitro and in vivo

The studies on the interactions between tumor and endothelial cells during the metastatic cascade require the elaboration of the experimental approaches that comprehensively address the hallmarks and mediators of endothelial activation. In vitro techniques enable the elucidation of the crosstalks between the circulating cancer and endothelial cells at the single-cell level. Transwell assays, based on the monitoring of cancer cell transmigration through the microporous membranes covered with endothelial layers, enable the quantification of endothelial contribution to the diapedesis of cancer cells. They are complemented by the analyses of endothelial permeability (electrical conductance or fluorochrome diffusion), which help to quantify endothelial integrity during the paracellular diapedesis of cancer or immune cells [155]. However, they do not give insight into the hallmarks of endothelial activation at the single-cell level, nor they visualize the subcellular processes that induce this process. Flow chamber assays in the microfluidic systems provide a setup for a more precise control of shear stress and flow rates inflicted on leukocytes or other rolling cells [156]. Concomitantly, several approaches have been proposed to trace the behavior of single circulating cells in vivo [157–162]. Epifluorescence intravital video microscopy (IVM) of blood vessels is an established method to evaluate the behavior of circulating cells and their interactions with endothelial layers. Whereas these approaches give insight into cell behavior in nearly physiological conditions, they hardly allow for the high-resolution analyses of the subcellular hallmarks of endothelial activation and its underlying mechanisms. This gap has been filled by the experimental approach based on the high-resolution imaging of the behavior of single cancer and endothelial cells in cocultures [12].

The coculture model gives a real-time insight into the endothelial barrier function during the diapedesis of cancer cells; in particular, it visualizes single endothelial cell properties related to their barrier function and to the collective mobilization of the endothelium. Cancer and endothelial cell behavior in cocultures can be monitored with time-lapse videomicroscopy and/or snapshotted by fluorescence microscopy. Potential experimental endpoints include cell morphology, motility, organization of adhesion sites, cytoskeletal architecture and contractility, and so on; immunofluorescence studies can also be used to concomitantly address the mechanisms underlying this process, including the activation of signaling pathways, oxidative stress, and so on, whereas genome editing can be used to elucidate the involvement of the specific proteins in these processes [15, 163]. The additional applications of extracellular matrix proteins and immune cells can be used to mimic the basal lamina and local inflammation, respectively [12, 14, 164]. These single-cell analyses can be complemented by en mass studies (reverse transcription quantitative polymerase chain reaction (RT–qPCR) and immunoblot) of the signaling pathways involved in the endothelial activation [12, 15]. We have successfully used this approach to scrutinize endothelial contribution to CTC diapedesis. It enabled (i) the unequivocal confirmation of the role of the endothelial activation in this process; (ii) the identification of the involved adhesive, paracrine, and juxtacrine pathways; and (iii) the estimation of the mechanisms underlying the contribution of immune cells to this process.

### At the crossroads of endothelial activation

Numerous mediators have been suggested to participate in the communication between endothelium and cancer cells during their diapedesis [14–20]. Adhesion-mediated intercellular signaling cooperates with CTC secretome to directly or indirectly activate endothelial cells, impair their barrier function, and facilitate CTC diapedesis. In addition, inflammation results in the locally enhanced production of humoral factors by immune and stromal cells, adding to the endothelial mobilization. Consequently, the interplay between paracrine and adhesion-dependent signaling pathways determines the activation of endothelial cells. It is further complemented by the direct gap junction mediated intercellular exchange of small metabolites to facilitate the transmigration of endothelial barriers by CTCs [14, 30, 165, 166].

#### Physical interactions

The adhesion of CTCs to the endothelium is a prerequisite of their effective diapedesis. Through the engagement of endothelial adhesion receptors, it prompts the cytoskeleton reorganization that reciprocally modulates the distribution and affinity of CAMs on endothelial surfaces. It also interferes with the stability of intercellular adhesion within endothelium and its integrity [167–170]. Its strength depends on the compatibility of cell adhesion receptors on the surfaces of CTCs and endothelial cells. Among the signaling systems involved, focal adhesion kinase (FAK) has been shown to integrate adhesive interactions between CTCs and endothelial cells with endothelial activation. Moreover, FAK inactivation (through the overexpression of dominant-negative FAK) decreased the adherence of cancer cells ability to the hepatic endothelium [171]. The quality of endothelial cell reactions to CTC signals is also modified by a physical milieu. Shear stress induces the adaptive responses of endothelium through the mechanisms responsive to the mechanoconductive properties of cytoskeleton [172]. Mechanosensors, integrated into protein complexes of focal contacts and adherens junctions, generate signals that modulate the functional status of the endothelium in the proximity of CTCs. The activation of down-stream mechanoresponsive cascades further affects the adhesive properties of endothelia, inducing the shifts of CAM levels and cytoskeletal rearrangements [173, 174] in cooperation with juxtacrine/paracrine signaling [30, 175, 176]. Thus, disturbances of the hemodynamic load may affect the pattern of endothelial activation and the efficiency of CTC diapedesis via the direct effect on endothelial integrity. By contrast, similar adhesive properties of cancer cells lineages, which differ in metastatic potential, indicate that the adhesion of tumor cells to endothelial layers is necessary but not sufficient for their enhanced diapedesis [177].

#### Paracrine signaling

Intersections of cytoskeleton with the signaling cascades activated by humoral factors provide the platform for the cooperation of adhesive and paracrine communication systems during the CTC-induced endothelial activation [77, 164]. Angioactive factors secreted by cancer cells can locally impair the endothelial barrier function via the induction of endothelial–mesenchymal transition [107, 178–180]. It locally impairs endothelial barrier function via the induction of endothelial cells motility and proliferation [78, 181]. The degradation of subendothelial matrices opens the windows for further subepithelial invasion of cancer cells via the undermining of the mechanical stability of endothelium. For instance, the stabilization of invadopodia prompts the secretion of proteases from cancer cells, including MMP2, MMP9, MT1-MMP, and urokinase plasminogen activator surface receptor (uPAR) [182, 183]. Notably, discrete lineages of cancer cells display unique patterns of secretome that selectively activate the adjacent endothelium to facilitate their diapedesis. For instance, human prostate cancer PC-3 cells secrete a much wider array of angioactive factors than their DU145 counterparts [14]. Importantly, the activated endothelial cells can further trigger autocrine loops to sustain and strengthen their own reactions to paracrine signaling from cancer cells. Such autocrine activation could be observed in the cocultures of lung cancer and endothelial cells, where the signals from cancer cells induce the local production of EGF. It impairs the endothelial barrier function but can also augment the invasiveness of cancer cells [15]. Alternatively, the paracrine signals from activated endothelium can strengthen the capillary arrest of cancer cells and sustain local inflammation via inducing blood coagulation, vascular remodeling, and collective tumor cell extravasation [95, 96, 184–186]. Collectively, a cooperative exchange of paracrine and adhesive stimuli between CTCs and local endothelia in premetastatic niches accounts for the activation of endothelium as a prerequisite for cancer cell extravasation.

#### Connexins and gap junctional coupling

Whereas the role of adhesive and paracrine signals in the communication between CTCs and endothelial cells in premetastatic niches is well recognized, the involvement of direct intercellular exchange of metabolites in CTC diapedesis is still a matter of controversy. This in particular concerns tubular nanotubes (TNTs), which have been observed to form cytoplasmic bridges between cancer and endothelial cells, but their biological significance remains obscure [187–190]. In turn, numerous studies confirmed the role of connexins and gap junctions in CTC diapedesis. Initially, the function of the gap junctions during the transmigration of immune cells was ignored; however the presence of connexin (Cx)43 in almost all types of immune cells and its upregulation observed in response to the inflammatory mediators, such as TNF-α and lipopolysaccharide (LPS), confirmed its crucial role in inflammation [191–195]. GJIC has been implicated in the direct communication of immune cells with the stroma and endothelium during the extravasation process [194]. In fact, a chemical inhibition of Cx43 channel function impairs the efficiency of cell diapedesis in numerous cellular systems [30, 165, 166, 196–201]. Mechanistic studies confirmed the intersections of GJIC with other mediators of endothelial activation. For instance, interferon gamma (IFN-γ) upregulates Cx43 in monocytes, the intensity of their GJIC with endothelial cells, and their diapedesis efficiency [192]. Moreover, the stimulation of leukocytes by TNF-α increased their adhesion to endothelial cells in a manner dependent on GJIC [201]. These notions were confirmed in vivo: TNF-α failed to increase the adhesion capacity of leukocytes and the efficiency of their diapedesis in mice lacking endothelial Cx43 expression.

The crucial role of connexins (Cx26, Cx32, and Cx43) and gap junctions in the metastatic cascade is illustrated by the correlation between the expression of connexins and the metastatic potential of cancer cells [202–205]. The notion on the corresponding function of Cx43 in in the diapedesis of CTCs has been experimentally confirmed by an enhanced transendothelial migration of breast cancer cells, following ectopic Cx43 expression [165]. In addition, the transfection of noninvasive melanoma cells with the gene encoding Cx26 significantly increased their metastatic potential and the ability to communicate through gap junctions with the endothelium and penetrate its monolayers [166]. Accordingly, we have shown the heterogeneity of Cx43 expression in prostate cancer cells, which correlated with their transmigration and diapedesis potential [196, 206]. Notably, Cx43 can affect cellular properties in GJIC-dependent and GJIC-independent ways [207]. The chemical inhibition of GJIC between breast cancer cells and endothelium significantly reduced their ability to penetrate endothelial layers [165]. Similarly, rat prostate AT-2 cells penetrated endothelial layers after establishing active gap junctions with endothelial cells, and this process was impaired by the inhibition of GJIC [196, 208]. By contrast, several studies show a GJIC-independent involvement of Cx43 in the diapedesis of cancer cells. The presence of Cx43 in the cell membrane increases the strength of the adhesion of breast cancer cells to the endothelium [30]. Using an experimental approach based on the confrontation of cancer and endothelial cells (cf. “At the crossroads of endothelial activation”), we showed that human prostate DU145 cells penetrated endothelial barriers in a Cx43-dependent/GJIC-independent manner. The efficiency of this process was sensitive to the changes of Cx43 levels, but remained insensitive to chemical GJIC inhibition [14]. These direct effects of Cx43 on the diapedesis of cancer cells were accompanied by its indirect Cx43-dependent modulation via the induction of epithelial-mesenchymal transition [196] and Cx43 accumulation on endothelial surfaces [14]. Collectively, the interplay between the paracrine signaling, adhesive interactions, and gap junctional communication at the interfaces of cancer and endothelial cells determines endothelial activation and the efficiency of cancer cell extravasation [42, 61, 209].

## Heterogeneity of CTCs and local inflammation

Endothelial susceptibility to the signals that disrupt endothelial barriers depends on intrinsic and extrinsic factors. Intrinsically, the phenotypic heterogeneity of endothelial cells is a consequence of their different origin, developmental career, and secular phenotypic status [26, 27, 35]. Extrinsically, phenotypic traits of endothelial cells are modulated by the dynamics of the niches and by the local inflammation processes. Inflammatory microenvironment of the premetastatic niches has long been suggested to facilitate the local diapedesis of CTCs [95, 96, 135, 180, 185, 186, 210]. Conceivably, the shifts in the levels of endothelial Cx43 that accompany their local inflammatory activation may enhance their susceptibility to the Cx43-mediated signals from CTCs. Thus, the compatibility of endothelial and CTC phenotype, which determines the efficiency of endothelial activation, depends on local inflammatory reactions. They can also indirectly account for the correlation between Cx43 levels and metastatic potential of cancer cells.

Extrinsic control of endothelial sensitivity to the signals from CTCs is exemplified by the differential adhesive affinity of discrete tumor cell lineages to the different endothelial strains [211, 212]. It cooperates with intercellular paracrine loops [213, 214] to determine the preference of certain types of tumors for the formation of metastases in specific tissues [215] as predicted by the “seed and soil” hypothesis [210, 216]. For instance, the predilection of CTCs to gestate in the bone marrow results from the specific structure of blood vessels in bones, which are characterized by a “discontinuous” structure and the lack of basement membranes [215]. However, compatibility of CAMs on the surfaces of CTCs and endothelia and their compatible secretome adds to this affinity [154]. These mechanisms can be retrospectively studied via the comparative proteomic analyses of cancer cells derived from the secondary tumors. For instance, the secretome of human prostate cancer PC-3 cells (derived from prostate cancer metastases to bone) considerably differed from that of their brain-metastasis-derived DU145 counterparts [12]. Apparently, a very compact structure of brain capillaries, the presence of basement membranes and a dense network of surrounding pericytes and astrocytes [36] enforce the secretion of specific sets of paracrine and juxtacrine meditators on extravasating cancer cells. Differences between discrete lineages of prostate cancer cells may thus illustrate phenotypic heterogeneity of CTCs and different vectors of selective forces that govern the formation of secondary tumors in different tissues. It prompts the selective gestation of their subclones in specific organs, according to the phenotypic compatibility of CTCs and local endothelia. Different diapedesis strategies employed by heterogeneous populations of CTCs may account for their heterogeneous niche preferences.

The phenotypic heterogeneity of endothelial cells and the size and type of blood vessels cooperate with the crosstalks between CTCs, immune, and endothelial cells to determine the tissue-specific pattern of CTC diapedesis [216, 217]. Local inflammation modulates the quality and quantity of endothelial surface complexes to facilitate the endothelial activation by the combined signals from immune and cancer cells. Furthermore, the shifts in Cx43 expression may account for these effects [12, 163]. Last but not least, clustering of CTCs with neutrophils (cf. “Contribution of immune system to the diapedesis of cancer cells” and [218]) provides the platform for the intercellular signaling between aggregated cells during the diapedesis [120]. The crosstalk between CTCs and platelets [219] enhances the metastatic potential of cancer cells [143, 220], in particular, their ability to extravasate in specific regions—in this case, in the regions of the local inflammation. Paracrine loops between stromal, endothelial, immune, and cancer cells concomitantly increase the invasive potential of cancer cells, providing the complementary route of enhancing the efficiency of cancer cells’ diapedesis [152]. Notably, such loops can also protect tumor cells from immune clearance at metastatic niches by creating the immunosuppressive microenvironment. For instance, T_regs_, MDSCs, and M2-polarized macrophages inhibit CD8^+^ T cell and NK cell cytotoxicity, thereby enabling the survival of extravasated tumor cells in colonized niches [221]. Tissue resident invariant natural killer T (iNKT17) cells can participate in this process via the IL-22/aminopeptidase N-dependent induction of endothelial permeability [222].

## Endothelial activation as a therapeutic target in tumor therapy

The significance of endothelial activation for the diapedesis of cancer cells suggests the potential of vasoactive drugs as agents that would complement regular chemotherapeutic approaches to reduce the risk of malignant cancer dissemination. Therapeutic strategies designed to inhibit tumor cell extravasation focus on two principal processes: (i) the modulation of CTC interactions with the endothelium and (ii) the improvement of endothelial barrier function [169, 190, 223–225]. The inhibition of endothelial CTC adhesion and transmigration can be achieved via targeting cell adhesion molecules (CAMs: integrins, selectins, and ICAM-1) [169]. For instance, uproleselan (GMI-1271), which is an E-selectin antagonist, shows promise in preventing leukemic cell adhesion to vascular endothelium [226]. In turn, modes of the therapeutic effects of etaracizumab (anti-α_v_β_3_ integrin Ab) were seen in phase I/II tests [227, 228]. CTC infiltration into surrounding tissues can also be inhibited by strengthening the endothelial barriers. In this context, the Tie1-targeting antibody has shown potential in preclinical models [229]. By contrast, anti-VEGF monotherapies gave limited clinical effects, which are limited to several tumor types [230–232]. For example, phase III clinical trials (e.g., AVADO, RIBBON-1) failed to confirm survival benefits of Bevacizumab (Avastin) and revealed the substantial adverse events of its application, including arterial thromboembolic events, severe hypertension, hemorrhage, and gastrointestinal perforation. In several other cases, these treatments were suggested to promote metastatic dissemination [233–236]. By inducing hypoxia within the tumor microenvironment, bevacizumab can apparently activate hypoxia-inducible factors (HIFs), which upregulate prometastatic genes and promote epithelial–mesenchymal transition (EMT) [236]. This can lead to increased tumor cell motility, invasion into surrounding tissues, and dissemination to distant organs.

The adverse effects of antiangiogenic strategies enforce a number of requirements on newly elaborated therapeutic agents, starting from their low systemic toxicity, through the specificity of their vasoactive effects, up to the negligible systemic adverse effects. This prompted research on natural plant compounds as agents that may improve the stability of endothelial barriers in the proximity of CTCs. For instance, rutin and diosmin have long been used to stabilize varicose veins [237–240]. It was shown that rutin, a natural flavonoid found in various fruits and vegetables, is able to protect endothelial barrier integrity during the diapedesis of leukocytes [238]. Rutin significantly reduced LPS-induced endothelial permeability, the expression of cell adhesion molecules (CAMs), and leukocyte adhesion and migration, both in vitro and in vivo. The protective role of rutin against hyperpermeability was also confirmed in an in vitro model of diabetic nephropathy, where hyperglycemia-induced disruption of the renal endothelial barrier was inhibited by rutin through the downregulation of the RhoA/ROCK signaling pathway [237]. Diosmin regulates endothelial permeability by strengthening tight junctions between endothelial cells and reducing inflammation-induced barrier disruption. It inhibits the expression of VEGF [240] and proinflammatory cytokines [241], which are key drivers of increased vascular permeability. However, it remains unknown whether these substances can also be applied to impair endothelial activation during CTC diapedesis.

By contrast, the vasoactivity of fenofibrate, accompanied by its cytostatic, proapoptotic, and anti-invasive activity in cancer systems and negligible side-effects, attracted our attention to its application as a potential inhibitor of CTC diapedesis. Notably, the application of this peroxisome proliferator-activated receptor alpha (PPARα) activator in hyperlipidemia has been approved by public health agencies, including the Food and Drug Administration (FDA), and its pharmacokinetics are well described [242, 243]. Numerous studies have revealed the inhibitory effect of fenofibrate on cancer development and its potential as the metabolic blocker in the combined cancer therapies [244–253]. Recently, we demonstrated that fenofibrate interferes with the drug-resistance of prostate cancer cells most probably via the interference with their energy metabolism (oxidative phosphorylation (OXPHOS)) and ATP production [254].

Apparently, fenofibrate efficiently interferes with the endothelial activation during cancer cell diapedesis [12, 15]. An experimental approach based on the cocultures of endothelial and cancer cells (cf. “Modeling endothelial activation in vitro and in vivo”) enabled us to visualize augmenting effects of fenofibrate on the endothelial barrier function. The monitoring of single endothelial cell properties related to their barrier function have revealed that fenofibrate impairs the susceptibility of endothelium to the activating signals from prostate [12] and lung cancer cells [15]. This effect was exerted through the inhibitory effect on the secretion of paracrine simulators in cancer cells, the impairment of GJIC between cancer and endothelial cells, and the ROS-dependent adhesion of endothelium to the underlying ECM [12, 15, 163]. Consequently, fenofibrate interferes with CTC-induced collective mobilization of the endothelium. These data show a new, previously ignored application of fenofibrate and, potentially, other vasoactive drugs in cancer treatment. They indicate the application of fenofibrate (and potentially of other vasoactive substances such as diosmin and rutin) for the interference with the “escape” strategy of CTCs and with the gestation of metastasis-initiating cells in premetastatic niches. It corresponds to the recently proposed cancer treatment strategies targeted at normal cells. Even though fenofibrate can also induce stem cell-dependent microevolution of drug-resistance [255], fenofibrate-induced impairment of the endothelial activation during cancer cell diapedesis indicates that its antimetastatic potential may prevail over the adverse effects related to the microevolution of tumor drug resistance.

## Summary and outlook

During cancer progression, the diapedesis of CTCs determines the venue of secondary tumor development and the formation of secondary tumors. It shares several characteristics with immune cell extravasation. However, the dimensions and limited deformability of CTCs makes the impairment of endothelial barrier function a sine qua non condition for the further gestation of CTCs in premetastatic niches. Consequently, the local activation of endothelium is decisive for the colonization of distant organs by CTCs. The sensitivity of local endothelium to paracrine, adhesive, and juxtacrine stimuli generated by CTCs in the premetastatic niches is determined by their compatibility with endothelial communication systems, adding to the image of cancer as a communication disease. The tissue-specificity of endothelial phenotypes and its consequences for the quality and quantity of adhesive, paracrine, and juxtacrine communication routes in metastatic niches underline the predilection of certain types of cancer to the specific organs. Thus, tissue-specific routes of endothelial activation follow and add to the “seed and soil” scheme of cancer development. Further research is necessary to fully elucidate the links between the local endothelial phenotype and the predilection of particular tumors to generate metastases in specific organs.

Similarly, the research must be intensified on the contribution of immune and stromal cells to endothelial preconditioning and activation during CTC diapedesis. The data on the links between inflammation and circulation disorders (such as atherosclerosis) indicate that local the chronic inflammation may be guiding the endothelial reprogramming toward the states permissive for CTC diapedesis. This process can be augmented by the activation of stromal cells, including the fibroblasts that otherwise have been implicated in the promotion of primary tumors [8]. In fact, the links between fibrosis and cancer have recently been extensively studied [256–261]. Collectively, these observations justify the intensification of the research on anticancer regimens that would target cancer-associated normal cells. Apart from anti-inflammatory and antifibrotic regimens, the crucial role of endothelial cells in CTC diapedesis pinpoints the systemic stabilization of endothelial barriers as the auxiliary aim in combined cancer therapies.

Diapedesis is a bottleneck of metastatic cascade; therefore, the augmentation of endothelial barrier function may delay the formation of metastases, improving the living standard of patients with diagnosed cancer. However, cancer is a microevolutionary disease and the majority of cancer-related deaths is related to the microevolution of invasive cancer cell lineages. The augmentation of endothelial barrier function may increase the selective pressure on CTCs, prompting the “survival of the fittest” cells and enhancing the malignancy of secondary tumors. Striking differences between diapedesis strategies employed by CTCs may additionally limit the efficiency of this strategy. Finally, the routes of systemic cancer cell dissemination overlap with the mechanisms of immune cell diapedesis. Therefore, the identification of cancer- and immune cell-specific routes of endothelial activation during diapedesis is another milestone on the way to the establishment of new therapeutic strategies that would complement (rather than replace) traditional cancer treatment regimens.

## Data Availability

Data sharing is not applicable to this manuscript as no datasets were generated or analyzed during the current study.

## Abbreviations

ACSL4: Acyl-coenzyme A synthetase long-chain family member 4
BBB: Blood–brain barrier
BMDCs: Bone-marrow-derived cells
CAFs: Cancer-associated fibroblasts
CaM: Calmodulin
CAMs: Cell adhesion molecules
CSCs: Cancer stem cells
CSF-1: Colony stimulating factor 1
CTCs: Circulating tumor cells
Cx: Connexin
ECH1: Enoyl-CoA hydratase 1
ECM: Extracellular matrix
EGF: Epidermal growth factor
EMT: Epithelial–mesenchymal transition
ERK: Extracellular signal-regulated kinases
FAK: Focal adhesion kinase
FDA: Food and Drug Administration
GJIC: Gap junctional intercellular communication
HIF: Hypoxia-inducible factors
IL: Interleukin
iNKT17: Invariant natural killer T
IVM: Intravital microscopy
JAMs: Junctional adhesion molecules
LBRC: Lateral border recycling compartment
LFA-1: Lymphocyte function-associated antigen 1
LOXL_2_: Lysyl oxidase-like 2
LPS: Lipopolysaccharides
Mac-1: Macrophage-1 antigen
MCP-1: Monocyte chemoattractant protein-1
MDSCs: Myeloid-derived suppressor cells
MLC: Myosin light-chain
MLCK: Myosin light-chain kinase
MMPs: Matrix metalloproteinases
NETs: Neutrophil extracellular traps
OXPHOS: Oxidative phosphorylation
PSGL-1: P-selectin glycoprotein ligand-1
PECAM-1: Platelet endothelial cell adhesion molecule
PI3K: Phosphatidylinositol 3-kinases
PMN: Premetastatic niche
PPARα: Peroxisome proliferator-activated receptor alpha
PUFAs: Polyunsaturated fatty acids
ROCK: Rho-associated kinase
ROS: Reactive oxygen species
TAMs: Tumor-associated macrophages
TEM: Transendothelial migration
TGF-β: Transforming growth factor β
TNF-α: Tumor necrosis factor alpha
TNTs: Tunneling nanotubes
uPAR: Urokinase plasminogen activator surface receptor
VCAM: Vascular cell adhesion protein 1
VEGF: Vascular endothelial growth factor
VLA-1: Very late antigen-1
